# Supplementary material for: DNA Methylation in Pituitary Adenomas: A Scoping Review
Source: Int J Mol Sci. 2025 Jan 10;26(2):531. doi: 10.3390/ijms26020531 (PMC11765255; doi:10.3390/ijms26020531)
Supplement: Supplementary file 1 [file ijms-26-00531-s001.zip › Table S1. Digestion analysis table.pdf]

Table S1. Digestion-restrictied analysis.

Author / Year	Aim	Sample Size	Key Findings
Hoi et al. (1) / 1988	Investigate the potential genetic basis for pituitary adenoma hormonal hypersecretion and autonomous growth	Two patients harboring a prolactinoma and an acromegalic adenoma	Hypomethylated GH gene in some human pituitary adenomas may be partially responsible for the hypersecretory state
Huttner et al. (2) / 1994	GH gene hypomethylation in human pituitary somatotropinomas	7 samples (5 PitNETs and 2 normal)	No alterations found in GH gene promotor region, but the gene appears to be hypomethylated
Woloschak et al. (3) / 1997	Primary human pituitary adenomas for evidence of p16 gene methylation	Pituitary tumor specimens from 20 patients	Methylation of the CpG island of the p16 gene is the predominant mechanism of p16 gene inactivation in these tumors
Simpson et al. (4) / 2000	Methylation status of the CpG island contained within the promoter region of the RB1 gene	Twelve sporadic, non-functional pituitary tumors (4 non-invasive and 8 invasive) and 18 somatotropinomas (11 noninvasive and 7 invasive)	A significant association between loss of pRb expression and methylation of the CpG island within the RB1 promoter region
Ruebel et al. (5) / 2001	Analysis of a group of pituitary tumors for p16 gene methylation and protein expression	24 frozen pituitary samples (21 PitNETs, 3 normal)	Analysis confirmed the high level of hypermethylation in null cell adenomas
Newell-Price et al. (6) / 2001	Patterns of methylation of POMC in normal and neoplastic tissue	6 samples. One ACTH PitNET and one NFPA.	POMC CpG island promoter is a powerful means of repressing expression
Picard et al. (7) / 2007	Hypothesized that an imprinting dysregulation of Gsa transcript in somatotroph tumors	11 pituitary adenoma samples	Partial methylation of exon 1A DMR and poor expression of exon 1A, inducing a higher expression of Gsa.

	could impact tumoral phenotype		
Raverot et al. (8) / 2010	Aggressive PitNETs and pituitary carcinomas correlated with MGMT promoter methylation	Four with PRL-secreting tumors (three carcinomas and one aggressive tumor) and four with ACTH tumors (two carcinomas and two aggressive tumors)	MGMT status as a poor predictor of outcome for treatment with Temozolomide.

1. Hoi SU, Kelley P, Lee WH. Abnormalities of the human growth hormone gene and protooncogenes in some human pituitary adenomas. Molecular Endocrinology. 1988;2(1):85-9.
2. Huttner A, Adams EF, Buchfelder M, Fahlbusch R. Growth hormone gene structure in human pituitary somatotrophinomas: Promoter region sequence and methylation studies. Journal of molecular endocrinology. 1994;12(2):167-72.
3. Woloschak M, Yu A, Post KD. Frequent inactivation of the p16 gene in human pituitary tumors by gene methylation. Molecular Carcinogenesis. 1997;19(4):221-4.
4. Simpson DJ, Hibberts NA, McNicol AM, Clayton RN, Farrell WE. Loss of pRb expression in pituitary adenomas is associated with methylation of the RB1 CpG island. Cancer Research. 2000;60(5):1211-6.
5. Ruebel KH, Jin L, Zhang S, Scheithauer BW, Lloyd RV. Inactivation of the p16 gene in human pituitary nonfunctioning tumors by hypermethylation is more common in null cell adenomas. Endocrine Pathology. 2001;12(3):281-9.
6. Newell-Price J, King P, Clark AJ. The CpG island promoter of the human proopiomelanocortin gene is methylated in nonexpressing normal tissue and tumors and represses expression. Molecular endocrinology (Baltimore, Md). 2001;15(2):338-48.
7. Picard C, Silvy M, Gerard C, Buffat C, Lavaque E, Figarella-Branger D, et al. Gsα overexpression and loss of Gsα imprinting in human somatotroph adenomas: Association with tumor size and response to pharmacologic treatment. International Journal of Cancer. 2007;121(6):1245-52.
8. Raverot G, Sturm N, De Fraipont F, Muller M, Salenave S, Caron P, et al. Temozolomide treatment in aggressive pituitary tumors and pituitary carcinomas: A French multicenter experience. Journal of Clinical Endocrinology and Metabolism. 2010;95(10):4592-9.
