# Supplementary material for: DNA Methylation in Pituitary Adenomas: A Scoping Review
Source: Int J Mol Sci. 2025 Jan 10;26(2):531. doi: 10.3390/ijms26020531 (PMC11765255; doi:10.3390/ijms26020531)
Supplement: Supplementary file 1 [file ijms-26-00531-s001.zip › Table S2. Chipbased methylation table.pdf]

Table S2. Chip-based methylation.

Author/ Year	Aim	Sample size	Key findings	Addtional
Duong et al. (1) / 2012	Methylation profile of 27 578 CpG sites spanning more than 14 000 genes in each of the major pituitary adenoma subtypes	7 GH-secreting tumors, 6 corticotrophinomas, 6 prolactinomas (PRL), and 13 non-functioning (NF) adenomas	First and unbiased survey of the pituitary tumor epigenome across different adenoma subtype	
Ling et al. (2) / 2014	DNA methylation alterations between invasive and noninvasive PAs subtypes	24 patients with surgically-resected PAs	DNA methylation analysis of key candidate genes may potentially be used to complement PA current histopathology subtype classification systems	
Gu et al. (3) / 2016	DNA methylation differences between invasive and noninvasive nonfunctioning PAs	19 PAs, 12 adenomas were included in the discovery cohort, and 7 adenomas were included in an independent cohort	Epigenetic modification of key gene substrates might partially account for the invasion of nonfunctioning PAs	
Kober et al. (4) / 2018	The role of aberrant methylation at particular loci for gene expression in PAs	31 gonadotroph NFPAs, two NFPAs that were positive for gonadotropins (FSH, LH, a-subunit), as well as TSH, and one null-cell adenoma	Invasiveness-related aberrant epigenetic upregulation of ITPKB and downregulation CNKSR1 in invasive NFPAs	
Boresowicz (5) / 2018	Incidence of TERT abnormalities and to assess their role in telomere lengthening in PAs	Tissue samples from 101 patients	Telomerase abnormalities do not play any special role in pathogenesis of pituitary tumors	

Johann et al. (6) / 2018	Characterize molecular alterations of sellar region ATRTs in adults as compared to pituitary adenomas	47 pituitary adenomas were evaluated	Sellar region ATRTs in adults form a clinically distinct entity with a different mutational spectrum	
Salomon et al. (7) / 2018	DNA methylation data were generated from the three major subtypes of pituitary adenomas	48 patients	DNA methylation alterations play a major role in the disease etiology	
Kober et al. (8) / 2019	DNA methylation in the misregulation of gene expression in gonadotroph NFPAs	32 patients	Genes with aberrant methylation in pituitary tumors STAT5A, RHOD, GALNT9, RASSF1, CDKN1A, TP73, STAT3 and HMGA2. FAM163A, HIF3A, and PRSS8, were hypermethylated in NFPAs	
Cheng et al. (9) / 2019	Integrated analyses of paired whole-genome DNA methylation and gene expression in PA	Retrospectively enrolled 68 patients	The methylation and expression levels of PHYHD1, LTBR, MYBPHL, C22orf42, PRR5, ANKDD1A, RAB13, CAMKV, KIFC3, WNT4 and STAT6 play a pivotal role in the invasive behavior of NFPA	
Neou et al. (10) / 2019	A molecularly unbiased classification, further deciphering the pathways responsible for	The methylome of 86 PitNETs of all types	Identified three groups associated with tumor type and secretion, in particular	

	tumorigenesis from a single set of PitNETs		POU1F1/PIT1-lineage tumors showed global hypomethylation	
Cheng et al. (11) / 2020	DNA methylation and expression parameters to evaluate the regrowth of NFPA	71 patients diagnosed with NFPA	6 of 13 genes (FAM90A1, ETS2, STAT6, MYT1L, ING2 and KCNK1) were considered as potential biomarkers associated with the regrowth of NFPA	
Boresowicz et al. (12) / 2020	CpGs located in miRNA genes that have differential methylation levels in gonadotroph PitNETs	34 PitNETs and five samples of normal pituitary	Epigenetic regulation and changes of miRNA expression play a significant role in pathogenesis of PitNETs	
Taniguchi-Ponciano et al. (13) / 2020	Identify the cellular pathways involved in their tumorigenesis	6 non-tumoral pituitaries and 42 PA	A divergent origin of PA, which segregates transcriptomically into three distinct clusters that depend on the specific transcription factors	
Wei et al. (14) / 2020	Activated and inhibited pathways and related key genes in hpNFPAs versus NFPAs	8 snap frozen NFPA specimens, including 4 hpNF- PAs and 4 NFPAs	DNA methylation and gene expression patterns of 2 highly proliferative NFPAs that occurred at young ages were noticeably distinct with other 6 NFPAs	
Mosella et al. (15) / 2021	Identify, characterize, and validate methylation-	DNA methylation data from PitNETs from three	Methylation signatures distinguished PitNETs	

	based signatures that define PitNETs according to clinicopathological features	independent institutions and from our cohort at the Hermelin Brain Tumor Center (n=23)	by adenohypophyseal cell lineages	
Schmid et al. (16) / 2021	Molecular differences between the three histologic types using DNA methylation analysis	47 neoplasms of the posterior pituitary gland	DNA methylation differences among tumors of the poste- rior pituitary are only subtle	
Nadaf et al. (17) / 2021	Use generated data to gain insights into the initiation and development of PitBs by identifying pathways differentially altered	A total of 64 tumor and normal FFPE tissues	PitB samples formed a distinct cluster separate from the various pituitary adenoma subtypes	
Hagel et al. (18) / 2021	12 double PAs (DPA) with diverse hormone profile were investigated in regard to DNA methylation profile	Twelve cases were identified among 3654 surgical specimens of adenoma	Global DNA methylation profiling may yield additional information in lesions that appear as null-cell adenomas immunohistochemically	
Asuzu et al. (19) / 2022	A mechanism of tumorigenesis with therapeutic implications for CD	Three tumors investigated for methylation (EPIC)	There may also exist histone modifications that contribute to the pathogenesis of wild-type CD that were not captured by DNA methylation approaches	
Dottermusch et al. (20) / 2022	The role of epigenomic analyses in the diagnostic workup of a challenging sellar lesion	57-year-old male	Exemplifies benefits and limitations of epigenomic analyses in molecular diagnostics	Case report

			of posterior pituitary neoplasms	
Hickman et al. (21) / 2022	A functioning corticotroph tumor with admixed adrenocortical cells and provide novel methylation profiling data	A 33-year-old man	The methylation profile of this tumor was unique but placed within the T-SNE plots adenohypophyseal entities with the closest match being corticotroph tumours.	Case report
Giuffrida et al. (22) / 2022	Correlate the methylation status of NFPAs and GH-omas with their epidemiological and clinicopathological features	Twenty-one PA samples (11 GH-omas, 10 NFPAs)	C7orf50, GNG7, and BAHCC1 genes, which have been found to be methylated, in pituitary tumor biology	
Hallén et al. (23) / 2022	Whether DNA methylation pattern differs in NFPAs between patients with residual adenoma with postoperative progression	28 tumors from the reintervention group and 21 tumors from the radiologically stable group	Methylation patterns associated with clinically significant tumor growth requiring reintervention	
Silva-Júnior et al. (24) / 2022	Methylome and transcriptome analysis of the 3 major subtypes of surgically resectable PitNETs	77 patients: (46 NFPT and 31 functioning pituitary tumors [FPT]	Methylome and transcriptome data resulted in 3 clusters, which were associated with each and with 2017 and 2022 WHO classifications	

Aydin et al. (25) / 2022	Evaluate the molecular profiling of NF-PitNETs at three biological levels	34 NF-PitNET samples (and 6 normal pituitary glands	Proposed hub proteins including DCC, DLG5, ETS2, FOXO1, HBP1, HMGA2, PCGF3, PSME4, RBPMS, RREB1, SMAD1, SOCS1, SOX2, YAP1, ZFHX3	
Herrgott et al. (26) / 2022	Differentiate PitNETs from OPD through analysis of LB specimens	PitNETs (n = 37)	PitNETs release DNA methylation markers in the serum/plasma	
Santana-Santos et al. (27) / 2022	The Northwestern Medicine (NM) classifier of CNS tumors was developed and validated	3905 central nervous tumor samples. 2801 samples were used in the original classifier training, and 1104 were used for validation.	Whole-genome methylation profiling of brain tumors for clinical testing has been developed and validated	
Tucker et al. (28) / 2023	Validate the differential DNA methylation and related MAX protein expression profiles between NFPA and GHPA	52 surgically resected tumors (37 NFPA, 15 GHPA)	MAX transcription factor binding sites are globally hypomethylated and demonstrate increased accessibility for transcription factor binding in GHPA compared to NFPA	
Galbraith et al. (29) / 2023	The clinical utility of DNA methylation for primary diagnosis of brain tumors	1921 primary CNS tumors	The diagnosis of meningioma, schwannoma, and pituitary adenoma, DNA methylation is of limited diagnostic or prognostic value	

Kober et al. (30) / 2023	Genome-wide DNA methylation patterns in somatotroph tumors	Forty-eight tumor samples	DNA methylation profile between three molecular subtypes are also undeniable	
Feng et al. (31) / 2023	DNA methylation analysis, in PPETS tumors and the comparing cohort	15 (posterior pituitary tumors) PPT	PPETS and PPT tumors form a distinct molecular cluster	

1. Duong CV, Emes RD, Wessely F, Yacqub-Usman K, Clayton RN, Farrell WE. Quantitative, genome-wide analysis of the DNA methylome in sporadic pituitary adenomas. Endocrine-related cancer. 2012;19(6):805-16.
2. Ling C, Pease M, Shi L, Punj V, Shiroishi MS, Commins D, et al. A pilot genome-scale profiling of DNA methylation in sporadic pituitary macroadenomas: Association with Tumor invasion and histopathological subtype. PLoS ONE. 2014;9(4):e96178.
3. Gu Y, Zhou X, Hu F, Yu Y, Xie T, Huang Y, et al. Differential DNA methylome profiling of nonfunctioning pituitary adenomas suggesting tumour invasion is correlated with cell adhesion. Journal of Neuro-Oncology. 2016;129(1):23-31.
4. Kober P, Boresowicz J, Rusetska N, Maksymowicz M, Goryca K, Kunicki J, et al. DNA methylation profiling in nonfunctioning pituitary adenomas. Molecular and Cellular Endocrinology. 2018;473((Kober, Boresowicz, Rusetska, Siedlecki, Bujko) Department of Molecular and Translational Oncology, Maria Sklodowska-Curie Memorial Cancer Center and Institute of Oncology, Warsaw, Poland(Maksymowicz) Department of Pathology and Laboratory Diagnostics, Ma):194-204.
5. Boresowicz J, Kober P, Rusetska N, Maksymowicz M, Goryca K, Kunicki J, et al. Telomere Length and TERT abnormalities in pituitary adenomas. Neuroendocrinology Letters. 2018;39(1):49-55.
6. Johann PD, Bens S, Oyen F, Wagener R, Giannini C, Perry A, et al. Sellar Region Atypical Teratoid/Rhabdoid Tumors (ATRT) in Adults Display DNA Methylation Profiles of the ATRT-MYC Subgroup. The American journal of surgical pathology. 2018;42(4):506-11.
7. Salomon MP, Wang X, Marzese DM, Hsu SC, Nelson N, Zhang X, et al. The Epigenomic Landscape of Pituitary Adenomas Reveals Specific Alterations and Differentiates Among Acromegaly, Cushing's Disease and Endocrine-Inactive Subtypes. Clin Cancer Res. 2018.
8. Kober P, Boresowicz J, Rusetska N, Maksymowicz M, Paziewska A, Dabrowska M, et al. The role of aberrant DNA methylation in misregulation of gene expression in gonadotroph nonfunctioning pituitary tumors. Cancers. 2019;11(11):1650.
9. Cheng S, Xie W, Miao Y, Guo J, Wang J, Li C, et al. Identification of key genes in invasive clinically non-functioning pituitary adenoma by integrating analysis of DNA methylation and mRNA expression profiles. Journal of translational medicine. 2019;17(1):407.

10. Neou M, Villa C, Armignacco R, Jouinot A, Raffin-Sanson ML, Septier A, et al. Pangenomic Classification of Pituitary Neuroendocrine Tumors. Cancer Cell. 2020;37(1):123-34.e5.
11. Cheng S, Li C, Xie W, Miao Y, Guo J, Wang J, et al. Integrated analysis of DNA methylation and mRNA expression profiles to identify key genes involved in the regrowth of clinically non-functioning pituitary adenoma. Aging (Albany NY). 2020;12(3):2408-27.
12. Boresowicz J, Kober P, Rusetska N, Maksymowicz M, Paziewska A, Dabrowska M, et al. DNA Methylation Influences miRNA Expression in Gonadotroph Pituitary Tumors. Life (Basel, Switzerland). 2020;10(5).
13. Taniguchi-Ponciano K, Andonegui-Elguera S, Peña-Martínez E, Silva-Román G, Vela-Patiño S, Gomez-Apo E, et al. Transcriptome and methylome analysis reveals three cellular origins of pituitary tumors. Scientific Reports. 2020;10(1):19373.
14. Wei Z, Zhou C, Li M, Huang R, Deng H, Shen S, et al. Integrated multi-omics profiling of nonfunctioning pituitary adenomas. Pituitary. 2021;24(3):312-25.
15. Mosella MS, Sabedot TS, Silva TC, Malta TM, Dezem FS, Asmaro KP, et al. DNA methylation-based signatures classify sporadic pituitary tumors according to clinicopathological features. Neuro Oncol. 2021;23(8):1292-303.
16. Schmid S, Solomon DA, Perez E, Thieme A, Kleinschmidt-DeMasters BK, Giannini C, et al. Genetic and epigenetic characterization of posterior pituitary tumors. Acta Neuropathologica. 2021;142(6):1025-43.
17. Nadaf J, de Kock L, Chong AS, Korbonits M, Thorner P, Benlimame N, et al. Molecular characterization of DICER1-mutated pituitary blastoma. Acta Neuropathologica. 2021;141(6):929-44.
18. Hagel C, Schuller U, Flitsch J, Knappe UJ, Kellner U, Bergmann M, et al. Double adenomas of the pituitary reveal distinct lineage markers, copy number alterations, and epigenetic profiles. Pituitary. 2021;24(6):904-13.
19. Asuzu DT, Alvarez R, Fletcher PA, Mandal D, Johnson K, Wu W, et al. Pituitary adenomas evade apoptosis via noxa deregulation in Cushing's disease. Cell Reports. 2022;40(8):111223.
20. Dottermusch M, Rotermund R, Ricklefs FL, Wefers AK, Saeger W, Flitsch J, et al. The Diagnostic Impact of Epigenomics in Pituicyte-derived Tumors: Report of an Unusual Sellar Lesion with Extensive Hemorrhage and Necrotic Debris. Endocrine Pathology. 2022;33(3):411-3.
21. Hickman RA, Gionco JT, Faust PL, Miller ML, Bruce J, Page-Wilson G, et al. Pituitary corticotroph tumour with adrenocortical cells: A distinct clinicopathologic entity with unique morphology and methylation profile. Neuropathology and applied neurobiology. 2022;48(2):e12754.
22. Giuffrida G, D'Argenio V, Ferrau F, Lasorsa VA, Polito F, Aliquo F, et al. Methylome Analysis in Nonfunctioning and GH-Secreting Pituitary Adenomas. Front Endocrinol (Lausanne). 2022;13:841118.
23. Hallén T, Johannsson G, Dahlén R, Glad CAM, Örndal C, Engvall A, et al. Genome-wide DNA Methylation Differences in Nonfunctioning Pituitary Adenomas With and Without Postsurgical Progression. J Clin Endocrinol Metab. 2022;107(8):2318-28.

24. Silva-Junior R, Bueno AC, Martins CS, Coelli-Lacchini F, Ozaki JGO, Almeida ESDC, et al. Integrating Methylome and Transcriptome Signatures Expands the Molecular Classification of the Pituitary Tumors. J Clin Endocrinol Metab. 2023;108(6):1452-63.
25. Aydin B, Beklen H, Arga KY, Bayrakli F, Turanli B. Epigenomic and transcriptomic landscaping unraveled candidate repositioned therapeutics for non-functioning pituitary neuroendocrine tumors. Journal of Endocrinological Investigation. 2023;46(4):727-47.
26. Herrgott GA, Asmaro KP, Wells M, Sabedot TS, Malta TM, Mosella MS, et al. Detection of tumor-specific DNA methylation markers in the blood of patients with pituitary neuroendocrine tumors. Neuro Oncol. 2022;24(7):1126-39.
27. Santana-Santos L, Kam KL, Dittmann D, De Vito S, McCord M, Jamshidi P, et al. Validation of Whole Genome Methylation Profiling Classifier for Central Nervous System Tumors. Journal of Molecular Diagnostics. 2022;24(8):924-34.
28. Tucker DW, Pangal DJ, Du R, Gogia AS, Tafreshi A, Ruzevick J, et al. Validation of Myc-Associated Protein X (MAX) regulation in growth hormone secreting and nonfunctional pituitary adenoma. PLoS ONE. 2023;18(4 April):e0284949.
29. Galbraith K, Vasudevaraja V, Serrano J, Shen G, Tran I, Abdallat N, et al. Clinical utility of whole-genome DNA methylation profiling as a primary molecular diagnostic assay for central nervous system tumors - A prospective study and guidelines for clinical testing. Neuro-Oncology Advances. 2023;5(1):vdad076.
30. Kober P, Rymuza J, Baluszek S, Maksymowicz M, Nyc A, Mossakowska BJ, et al. DNA Methylation Pattern in Somatotroph Pituitary Neuroendocrine Tumors. Neuroendocrinology. 2024;114(1):51-63.
31. Feng J, Duan Z, Yao K, Gui Q, Liu X, Wang X, et al. Primary papillary epithelial tumor of the sella and posterior pituitary tumor show similar (epi)genetic features and constitute a single neuro-oncological entity. Neuro-Oncology. 2023;25(8):1487-97.
