# Supplementary material for: DNA Methylation in Pituitary Adenomas: A Scoping Review
Source: Int J Mol Sci. 2025 Jan 10;26(2):531. doi: 10.3390/ijms26020531 (PMC11765255; doi:10.3390/ijms26020531)
Supplement: Supplementary file 1 [file ijms-26-00531-s001.zip › Table S3. Additional papers.pdf]

Table S3. Additional papers.

Author/ Year	Aim	Sample Size	Key Findings	Addtional
Watanabe et al. (1) / 2010	ZAR1 non-promoter methylation using MassARRAY EpiTYPER	10 pituitary adenomas	Hypermethylation of the ZAR1 non-promoter was found to be a frequent event in pituitary adenomas	
Salehi et al. (2) / 2011	MGMT promoter methylation status in SS3 adenomas and pituitary carcinoma	12 SS3 pituitary adenomas and 10 pituitary carcinomas	MGMT promoter methylation occurs in a significant subset of pituitary carcinomas and SS3 adenomas but MGMT immunoreactivity is primarily negative	MS-MLPA study (methylation-specific multiplex ligation-dependent probe amplification)
Batisse et al. (3) / 2013	10 years' follow-up of a patient with silent GH aggressive pituitary tumor	A 47-year old male	GH tumor resistant to TMZ with unmethylated promotor for MGMT	
Garcia-Martinez et al. (4) / 2019	The role of methylation in gene expression and in the growth, invasiveness, and function of PitNETs	105 patients	Methylation status has a selective and limited effect on the growth and invasive behavior of PitNETs	MS-MLPA study
Szabó et al. (5) / 2020	Methylation and demethylation status of global DNA in PitNETs	45 patients with PitNET	The PitNET demethylation process correlated negatively with the proliferation rate	High-performance liquid chromatography–tandem mass spectrometry (LC-MS/MS method)

Rusetska et al. (6) / 2021	The methylation level of LINE-1 repetitive elements and its role in invasive in PitNETs	80 patients and 5 samples of normal human pituitary tissue	Invasive PitNETs are characterized by decreased DNA methylation at LINE-1 repetitive elements as compared to noninvasive tumors	Pyrosequencing study
Chang et al. (7) / 2022	The potential role of m6A and methyltransferase METTL3 during pathology of GH-PA tumorigenesis	16 GH-PAs, 25 nonsecreting PAs, and 6 normal pituitaries	The GH-PA–specific regulation of RNA m6A hypermethylation, orchestrated by overexpression of METTL3, is essential in regulation of cell growth or hormone secretion of GH-PA	m6A enzyme-linked immunosorbent assay and m6A sequencing (m6A-seq)
Ronsley et al. (8) / 2022	An unusual SMARCB1-altered tumor, originally diagnosed as atypical pituitary adenoma	One male	DNA methylation classifies the tumor better and shows patterns not showing up with usual testing.	
Barrantes-Freer et al. (9) / 2024	Epigenomic profiling of ectopic PitNET/adenomas	An 81-year-old female	Genome-wide DNA methylation analyses as a powerful tool to aid in the diagnostics of these challenging lesions	

1. Watanabe T, Yachi K, Ohta T, Fukushima T, Yoshino A, Katayama Y, et al. Aberrant hypermethylation of non-promoter zygote arrest 1 (Zar1) in human brain tumors. Neurologia Medico-Chirurgica. 2010;50(12):1062-9.
2. Salehi F, Scheithauer BW, Kros JM, Lau Q, Fealey M, Erickson D, et al. MGMT promoter methylation and immunoexpression in aggressive pituitary adenomas and carcinomas. Neuropathology and Applied Neurobiology. 2011;37(SUPPL. 1):17-8.
3. Batisse M, Raverot G, Maqdasy S, Durando X, Sturm N, Montoriol PF, et al. Aggressive silent GH pituitary tumor resistant to multiple treatments, including temozolomide. Cancer Investigation. 2013;31(3):190-6.
4. Garcia-Martinez A, Sottile J, Sa Nchez-Tejada L, Fajardo C, Camara R, Lamas C, et al. DNA Methylation of tumor suppressor genes in pituitary neuroendocrine tumors. Journal of Clinical Endocrinology and Metabolism. 2019;104(4):1272-82.
5. Szabo B, Nemeth K, Meszaros K, Szucs N, Czirjak S, Reiniger L, et al. Demethylation Status of Somatic DNA Extracted From Pituitary Neuroendocrine Tumors Indicates Proliferative Behavior. J Clin Endocrinol Metab. 2020;105(6).
6. Rusetska N, Kober P, Krol SK, Boresowicz J, Maksymowicz M, Kunicki J, et al. Invasive and noninvasive nonfunctioning gonadotroph pituitary tumors differ in dna methylation level of line-1 repetitive elements. Journal of Clinical Medicine. 2021;10(4):1-12.
7. Chang M, Wang Z, Gao J, Yang C, Feng M, Niu Y, et al. METTL3-mediated RNA m6A Hypermethylation Promotes Tumorigenesis and GH Secretion of Pituitary Somatotroph Adenomas. Journal of Clinical Endocrinology and Metabolism. 2022;107(1):136-49.
8. Ronsley R, Boue DR, Venkata LPR, Scott S, Shaikhouni A, Jones J, et al. An unusual case of atypical teratoid/rhabdoid tumor, initially diagnosed as atypical pituitary adenoma in a 13-year-old male patient. Neuro-Oncology Advances. 2022;4(1):vdac121.
9. Barrantes-Freer A, Braune M, Sandner B, Dottermusch M, Lindner D. Comparative epigenomics indicate a common origin of ectopic and intrasellar corticotroph pituitary neuroendocrine tumors/adenomas: a case report. Virchows Archiv. 2024((Barrantes-Freer, Braune) Paul-Flechsig-Institute of Neuropathology, University Hospital Leipzig, Leipzig, Germany(Sandner) Medical Department III - Endocrinology, Nephrology, Rheumatology, University Hospital Leipzig, Leipzig, Germany(Dottermusch) Instit).
