# Supplementary material for: DNA Methylation in Pituitary Adenomas: A Scoping Review
Source: Int J Mol Sci. 2025 Jan 10;26(2):531. doi: 10.3390/ijms26020531 (PMC11765255; doi:10.3390/ijms26020531)
Supplement: Supplementary file 1 [file ijms-26-00531-s001.zip › Table S4. Search string Embase S4.pdf]

Table S4. Search-string for Embase. 29th April 2024.

Step	Search String
1	Hypophysis tumor
2	exp Pituitary Neoplasms/ OR exp Pituitary Gland/
3	acth-secreting pituitary adenoma/ OR growth hormone-secreting pituitary adenoma/ OR prolactinoma/
4	exp acth-secreting pituitary adenoma/ OR exp growth hormone-secreting pituitary adenoma/
5	exp Pituitary Neoplasms/
6	exp Acromegaly/
7	Hypophysis tumor
8	Hypophysis tumo
9	2 OR 3 OR 4 OR 5 OR 6
10	(Epigenetics OR Epigenome OR Methylome OR DNA methylation OR CpG island).mp. [mp=title, book title, abstract, original title, name of substance word, subject heading word, floating sub-heading word, keyword heading word, organism supplementary concept word, protocol supplementary concept word, rare disease supplementary concept word, unique identifier, synonyms, population supplementary concept word, anatomy supplementary concept word]
11	(hypophys?al AND tumo?r*).mp. [mp=title, book title, abstract, original title, name of substance word, subject heading word, floating sub-heading word, keyword heading word, organism supplementary concept word, protocol supplementary concept word, rare disease supplementary concept word, unique identifier, synonyms, population supplementary concept word, anatomy supplementary concept word]
12	((hypophys?al AND tumo?r*) OR (hypophysi? AND tumo?r*) OR hypophysoma* OR (neoplastic AND pituitary*) OR (pituitary AND cell AND tumo?r*) OR (pituitary adj2 neoplasia) OR (pituitary adj2 neoplasm*) OR (pituitary adj2 tumo?r*)).mp. [mp=title, book title, abstract, original title, name of substance word, subject heading word, floating sub-heading word, keyword heading word, organism supplementary concept word, protocol supplementary concept word, rare disease supplementary concept word, unique identifier, synonyms, population supplementary concept word, anatomy supplementary concept word]
13	Expanded version: ((hypophys?al AND tumo?r*) OR (hypophysi? AND tumo?r*) OR hypophysoma* OR (neoplastic AND pituitary*) OR (pituitary AND cell AND tumo?r*) OR (pituitary adj2 neoplasia) OR (pituitary adj2 neoplasm*) OR (pituitary adj2 tumo?r*) OR (pituitary adj2 tumorigenesis) OR (tumo?r AND of AND the AND hypophysis) OR (tumo?r AND of AND the AND pituitary AND gland) OR (tumorigenesis AND of AND the AND pituitary AND gland) OR (pituitary AND adenoma*) OR (adenoma* AND of AND the AND pituitary AND gland) OR hypophyse adenoma* OR (hypophyseal AND adenoma*) OR (pituitary gland adenoma*) OR (secreting AND adenoma* AND of AND the AND pituitary AND gland) OR (secreting AND hypophyseal adenoma*) OR (secreting AND hypophysis adenoma*) OR (secreting AND pituitary adenoma*) OR ((acth adj2 pituitary) AND

Step	Search String
	adenoma*) OR ((acth adj2 pituitary) AND tumo?r*) OR (gonatroph adenoma*) OR (gonadotroph tumo?r*) OR (gonadotrop?ic adenoma*) OR gonadotropinoma* OR ((gh adj2 pituitary) AND adenoma*) OR ((gh adj2 pituitary) AND tumo?r*) OR (((growth hormone adj2 pituitary) AND adenoma*) OR (((growth hormone adj2 pituitary) AND tumo?r*) OR somatotroph adenoma*) OR (somatotropic adenoma*) OR (plurihormonal pituitary adenoma*) OR (lactotroph adenoma*) OR ((prl adj2 pituitary) AND adenoma*) OR ((prl adj2 pituitary) AND tumo?r*) OR ((prolactin adj2 pituitary) AND adenoma*) OR ((prolactin adj2 pituitary) AND tumo?r*) OR prolactinoma* OR (thyroid AND stimulating hormone AND secreting AND pituitary AND adenoma*) OR (thyroid AND stimulating hormone AND secreting AND pituitary AND tumo?r*) OR thyrotroph adenoma* OR ((thyrotropin adj2 pituitary) AND adenoma*) OR ((thyrotropin adj2 pituitary) AND tumo?r*) OR thyrotropinoma* OR ((tsh adj2 pituitary) AND adenoma*) OR ((tsh adj2 pituitary) AND tumo?r*) OR (non-functioning pituitary adenoma*) OR (nonfunctioning pituitary adenoma*) OR hypophyseal macroadenoma* OR (hypophysis AND macroadenoma*) OR (pituitary gland macroadenoma*) OR (pituitary macro-adenoma*) OR (pituitary macroadenoma*) OR hypophyseal microadenoma* OR (pituitary gland microadenoma*) OR (pituitary micro-adenoma*)).mp. [mp=title, book title, abstract, original title, name of substance word, subject heading word, floating sub-heading word, keyword heading word, organism supplementary concept word, protocol supplementary concept word, rare disease supplementary concept word, unique identifier, synonyms, population supplementary concept word, anatomy supplementary concept word]
14	9 OR 13
15	(epigenomics OR (dna AND methylation) OR (dna AND methylome) OR (deoxyribonucleic acid AND methylation) OR (dna AND hypermethylation) OR (dna AND hypomethylation) OR (methylated deoxyribonucleic acid) OR (methylated dna) OR (cpg island*) OR (cpg rich island*) OR epigenome OR methylome).mp. [mp=title, book title, abstract, original title, name of substance word, subject heading word, floating sub-heading word, keyword heading word, organism supplementary concept word, protocol supplementary concept word, rare disease supplementary concept word, unique identifier, synonyms, population supplementary concept word, anatomy supplementary concept word]
16	10 OR 15
17	14 AND 16
